# Forgive and Forget: Differences between Decisional and Emotional Forgiveness

**DOI:** 10.1371/journal.pone.0125561

**Published:** 2015-05-06

**Authors:** Stephanie Lichtenfeld, Vanessa L. Buechner, Markus A. Maier, Maria Fernández-Capo

**Affiliations:** 1 Department of Psychology, University of Munich, Munich, Germany; 2 Department of Basic Sciences, Universitat Internacional de Catalunya, Barcelona, Spain; University of Florida, UNITED STATES

## Abstract

To forgive and forget is a well-known idiom, which has rarely been looked at empirically. In the current experiment, we investigated differences between emotional and decisional forgiveness on forgetting. The present study provides the first empirical support that emotional forgiveness has a strong influence on subsequent incidental forgetting. Specifically, our results demonstrate that emotional forgiveness leads to substantially higher levels of forgetting in respect to offense relevant traits compared to both decisional forgiveness and no forgiveness. This provides evidence for our hypothesized effect that only individuals who have emotionally forgiven a transgression, and not those who just decided to forgive, subsequently forget offense relevant traits attributed to the transgressor.

## Introduction

“Forgive and Forget: Healing the Hurts We Don’t Deserve”, a trade book by Lewis Smedes [1], partially instigated a surge of research on forgiveness and it’s benefits for a person’s mental health and well-being [2]. However, while the title of the book suggests that forgiving and forgetting are strongly intertwined, their relationship has rarely been tested empirically. Thus, the focus of the present study is to investigate how different facets of forgiveness may influence forgetting.

Theoreticians and researchers have used several different definitions of forgiveness [3]. There seems to be a general consensus that forgiveness is a complex phenomenon [4], which entails cognitive [5], affective [6], behavioral [7], motivational [8], decisional [9], and interpersonal (e.g., [10]) components. However, there is disagreement whether empathy and the replacement of negative emotions with positive ones is a core aspect of forgiveness [11] or if the mere decision to forgiven is sufficient.

Worthington and colleagues [3,12] emphasize the role of emotion in the forgiveness process by distinguishing between decisional and emotional forgiveness. Decisional forgiveness is supposed to be a behavioral intention statement that one will eliminate revenge and avoidance and possibly restore interaction if the threat of future harm can be prevented. However, one may grant decisional forgiveness while still holding a grudge against the transgressor. This phenomenon is known in the literature as “hollow forgiveness” [10]. Emotional forgiveness is the replacement of negative, unforgiving emotions with positive, other-oriented ones [13] (for a review of empirical evidence in support of this distinction, see [14]). Likewise, emotion is considered an important aspect in several other models of forgiveness. For instance, Fitzgibbons [15] suggested that a clients’ intellectual decision to forgive is followed by emotional forgiveness, that is, when someone truly feels like forgiving the transgressor. Also, Enright and the Human Development Study Group [16] proposed that the forgiveness process incorporates the commitment to forgive the offender, which is usually a cognitive decision to forgive the other person, as well as an emotional forgiveness component, namely the decrease of negative and the increase of positive affect [17]. In their meta-analysis, Strelan and Covic [18] claim that several process models of forgiveness incorporate the decision to forgive or consider forgiving [15,16,19,20] as well as an understanding of, or empathy for, the offender [6,15,16,19–21]. Worthington and colleagues claim that “just as there is a distinction between decisional and emotional decision making, there may be a similar distinction between decisional and emotional forgiveness and processes” [22].

Considering the role of emotions in the decision making process, research in this field was dominated by the view that decision making is merely a rational process, which involves Bayesian maximization of expected utility, and emotions are only distracting individuals from rational decision making (see [23]). Only after the publication of Descartes’ Error by Antonio Damásio [24], who challenged the traditional ideas about the connection between emotions and rationality, the assumption that emotions are detrimental for decision making processes began to change. Nowadays, it is widely acknowledged that emotions serve adaptive functions because they prioritize certain goals and thereby mobilize energy and give direction to behavior [25–29].

In our view, just as decision making research has documented that behavioral choices can benefit from the joint interplay between emotional and cognitive processes, in the same way forgiveness might also benefit from an emotional commitment when forgiving another person. Thus, we argue it may be an important first step to decide to forgive a transgression, but in order to truly forgive one has to also emphasize and feel at peace with the transgressor.

While several theories incorporate decisional and emotional processes of forgiveness in their model, empirical evidence in respect to differences between these processes is rare. This is particularly true for experimental research studies. Hardly any study has manipulated emotional versus decisional forgiveness and investigated consequences of these different forgiveness processes. Thus, the aim of the present study is to investigate differences between decisional and emotional forgiveness on cognitive processes involved in the forgiveness process, namely forgetting. According to Fincham, Hall, and Beach [30] most definitions of forgiveness incorporate that “one becomes less motivated to think […] negatively in regard to the offender”. Thus, forgetting negative offense-relevant characteristics of an offender may be a hint that one feels at peace with a transgressor. Given that forgiveness in its actual sense requires that an individual emotionally forgives another person, we suppose that emotional forgiveness leads to greater forgetting of these characteristics as opposed to decisional forgetting.

Indirect evidence for the interrelation of forgiving and forgetting has been provided by Rhoades et al. [31], who investigated how being against forgiving the perpetrators (anti-forgiveness), being unsure about forgiveness (ambivalent), or trying to or having forgiven the perpetrators (pro-forgiveness) of the 9/11 attacks related to involuntary engagement in thinking and feeling about the event. Conforming to the supposition that forgiveness should lead to forgetting, they found that those who had either decided to try to forgive or who had already forgiven the attackers experienced less involuntary engagement, that is intrusive thoughts, physiological arousal, and rumination, than did both the ambivalent and anti-forgiveness groups. However, it has not been tested if those in the forgiveness condition only stopped actively engaging in thoughts about the event or indeed forgot details or aspects of the event. Moreover, forgiveness was measured rather than manipulated in this study, which does not allow for causal interpretations. Another recent study has examined the relationship between forgiveness and directed forgetting [32]. In this study participants were confronted with hypothetical situations (such as infidelity, slander, and theft) that they had never encountered before and were asked whether or not they would forgive such an offense. In a consecutive session, individuals were told to briefly summarize the associated offense, the consequence of the offense, and what the transgressor did to make amends (think condition) or were instructed to avoid thinking or saying anything about the associated scenario (no-think condition). In the final recall phase individuals were more likely to intentionally forget the forgiven scenarios in the no-think condition as compared to the unforgiven scenarios. Yet, this study investigated hypothetical offenses that subjects have never encountered before (for a claim that studies should focus on real life experiences, see [22]). Moreover, this study shows that suppressing information is easier for forgiven incidents as compared to unforgiven incidents, but it has not been examined if individuals forget unintentionally when they experience forgiveness toward a transgressor. Finally, in this study forgiveness has not been manipulated, but was assessed, and the researchers did not distinguish between different facets of forgiveness (decisional vs. emotional forgiveness).

In sum, the central aim of the present experiment was to investigate the causal relation between different facets of forgiveness (decisional vs. emotional forgiveness) and forgetting. Based on the adaptive function of forgiving and maintaining a benevolent relationship, we hypothesized that emotional forgiveness would cause individuals to incidentally forget traits of a transgressor that are associated with an offense.

## Materials and Methods

### Ethics Statement

The research reported herein was conducted at the LMU Munich and was approved by the ethics committee of the Department of Psychology, LMU Munich, in accordance with the ethical standards expressed in the Declaration of Helsinki. All participants gave verbal informed consent and were thoroughly debriefed. Verbal consent was considered to be sufficient, since it was ensured that data were stored and analyzed anonymously. The individuals’ verbal consent was obtained after reading the instruction to the experiments. The experimenter asked for the participant’s consent and emphasized that they will receive their credit also if they decided not to participate in this study. Participants were also told that they could stop and leave the experiment at any point of time. This consent procedure has been approved by the ethics committee.

### Participants

Forty-two undergraduate students (all female, mean age 22.3 years, SD = 7.8) at a German university participated for course credit. All participants were tested individually by an experimenter blind to participants’ condition and the experimental hypothesis.

### Materials

#### Scenarios

In accord with Worthington et al.’s [22] call for ecological validity that studies must employ tasks that mirror daily life, we developed two scenarios that were realistic offences experienced by students at a university. One scenario described a situation in which a group of students had to prepare a presentation for a class. The transgressor was described as showing very little engagement and leaving the work to the other students, so that the group received a worse grade than anticipated. The second scenario also described a situation in which a group of students had to prepare a presentation for a class and oneself has a very innovative idea how to run the presentation. When telling the professor about the plan and the professor likes it, the transgressor proclaims the idea to be hers and consequently gets a better grade than the rest of the group. The scenarios were tested in a pilot sample (n = 55), in which participants were asked to rate the scenarios according to their valence, arousal, and severity of the transgression. Results indicate that the scenario that describes a fellow student, who leaves the work to the other students is judged as being less negative (*t*(53) = 4.47, *p* < .001), less arousing (*t*(53) = 5.39, *p* < .001), and less severe (*t*(53) = 7.54, *p* < .001) as compared to the scenario that describes a student, who takes credit for another person’s idea. Thus, the two scenarios represent two different kinds of transgressions that vary in respect to their emotional strength and severity. This factor will thus be controlled in the subsequent analyses when testing our main hypotheses.

#### Trait words

Three experts selected 20 trait words that were characteristic of the person described in the scenario. For each scenario 10 of the 20 trait words were offence relevant (e.g., for the scenario describing a lazy person, who left all the work to the others, “lazy” was an offence relevant word, whereas for the scenario describing a person who took credit for another person’s idea, “egoistic” was an offence relevant word). Each trait word was relevant for one scenario, but not for the other. Thus, for half of the participants 10 of the trait words were offence relevant, while for the other half the other 10 trait words were offence relevant depending upon the scenario they had read. Scenarios were counterbalanced across conditions so that the results could not be attributed to word difficulty (e.g., lazy might be easier to recall than egoistic). The selected words were tested in the same pilot study as the scenarios. Participants were asked to indicate to what extend the trait adjectives would apply to the described person. T-Tests indicated that the trait words that were selected for the scenario that described a lazy person were judged as applying much more to a person described in this scenario than to the other one (*t*(53) = 8.82, *p* < .001). Likewise, trait words that were selected for the scenario that described a person, who takes credit for another person’s idea, were judged as applying much more to a person described in this scenario than to the other one (*t*(53) = 8.00, *p* < .001). In sum, the pilot data strongly supported that the selected words were offence relevant for one scenario but not the other, respectively.

### Procedure

The experiment was run using Eprime software version 2.0 (Psychology Software Tools, Inc., Pittsburgh, PA). Participants were asked to follow the instructions on the computer screen. At the beginning of the experiment, participants were provided with a description and illustration of one of two scenarios (counterbalanced across participants).

After reading the scenario, participants were told that several characteristics of the described person would be presented to them. The 20 trait words were presented for 1,000 ms in random order. Afterwards, participants were randomly assigned to one of three between-subjects conditions: the decisional forgiveness condition, the emotional forgiveness condition, or the control condition. Forgiveness was manipulated using an adapted version of Worthington et al.’s [33] manipulation procedure and participants were asked to follow the instructions for the next 45 seconds. In the decisional forgiveness condition they were asked to think of that person as a human being that misbehaved and to resolve not to pay her back and to behave positively and not negatively toward the offender. In the emotional forgiveness condition they were asked to wish that the offender experiences something positive or healing and to focus their feelings on empathy. In the control condition participants were asked to think about one’s own thoughts, feelings, and physical reactions in this situation and what they would think and how they would react in such an instance. Then, participants were asked to answer three filler questions related to the scenario.

After a 2-min distractor task (solving simple arithmetic problems), a free-recall test followed in which participants were instructed to write down as many of the previously presented traits of the person as possible for 30 seconds.

Finally, participants completed a brief demographics questionnaire, given their extra credit, and were debriefed and dismissed.

## Results

Memory performance was scored in terms of the absolute number of words recalled completely correctly. An additional analysis counting semantically very similar words as correct responses did not change the results, so we decided to retain the original and more objective criterion.

Preliminary analyses including scenario condition as a second between-subjects factor revealed a significant Scenario x Relevance Interaction (*F*(1, 36) = 4.91, *p* = .03, *η*_*p*_^*2*^ = .12) indicating that trait words such as “egoistic” were remembered better than trait words such as “lazy”. In respect to our main hypothesis no significant three-way interaction (Scenario x Relevance x Forgiveness Condition) was found suggesting that the effect of forgiveness on memory performance can be generalized across different scenarios. So, this variable was omitted from further consideration. Additionally, an analysis of variance (ANOVA) was performed and yielded no effect of condition on overall memory performance, *F*(2, 39) = .291, *p* = .75, indicating that participants did not differ in their overall memory performance.

To test our main hypothesis, that emotional forgiveness leads to forgetting of offense relevant traits a 3 (condition; between-subjects: emotional forgiveness, decisional forgiveness, control) × 2 (offense relevance; within-subjects: relevant, irrelevant traits) mixed-design ANOVA was performed on the correct memory of personality traits from the free-recall test.

**Fig 1:**
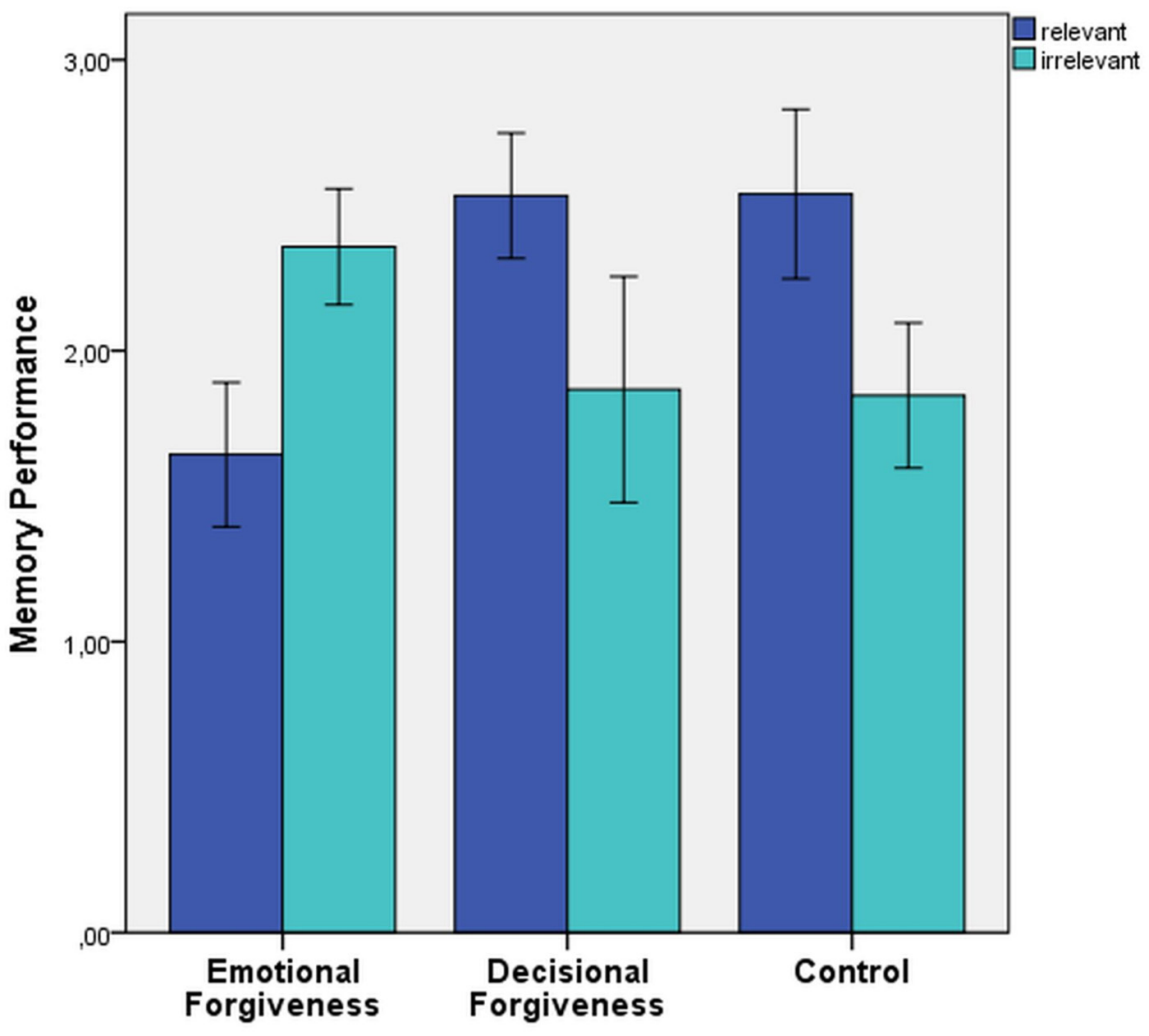
Memory performance as a function of forgiveness manipulation and relevance.

No main effect of offense relevance was found, *F*(1, 39) = 1.09, *p* = .30, indicating that overall irrelevant traits were remembered as well as relevant traits. More importantly, the 3 × 2 mixed-design ANOVA yielded a significant Relevance × Condition interaction was found, *F*(2, 39) = 5.12, *p* = .01, *η*_*p*_^*2*^ = .21 (Fig 1).

Tukey’s HSD test was used in conducting post hoc pairwise multiple comparison analyses. These analyses indicated that participants in the emotional forgiveness condition recalled significantly fewer offence relevant traits (*M* = 1.64, *SD* = .93) than those in the decisional forgiveness (M = 2.53, SD = .83), *p* < .05, *d* = 1.05, and the control condition (*M* = 2.54, *SD* = 1.05), *p* < .05, *d* = 0.94. In contrast, there were no differences between conditions in respect to offence irrelevant trait words (*F*(2, 39) = .937, p = .40). Specifically, participants in the emotional forgiveness condition recalled an equal amount of offence irrelevant traits (*M* = 2.36, *SD* = .74) as those in the decisional forgiveness (M = 1.87, SD = 1.51) and the control condition (*M* = 1.85, *SD* = .90).

In addition to the between group analyses reported above we also determined whether memory for relevant traits was enhanced as compared to memory for irrelevant traits separately within each group. A priori contrasts were performed showing that participants in the emotional forgiveness condition recalled significantly fewer relevant as compared to irrelevant traits, *t*(13) = 2.69, *p* = .02, *d* = 1.49 whereas participants in the decisional forgiveness condition, *t*(14) = -1.54, *p* = .15, *d* = .82, and the control condition, *t*(12) = -2.11, *p* = .06, *d* = 1.22, recalled comparable amounts of relevant and irrelevant traits.

## Discussion

The well-known idiom to “forgive and forget” suggests that you should not only forgive others for hurting you, but also forget that they ever hurt you. Tracing back to biblical verses such as Heb 8:12 (“For I will forgive their wickedness and will remember their sins no more.”) the term to “forgive and forget” enjoys a long history and is present in everyday language as well as in several titles of published research (e.g., [34–37]). However, despite the fact that the interrelation of forgiving and forgetting is widely acknowledged, there is scant empirical evidence for this assumption. The present study provides the first empirical support that specific subcomponents of forgiveness have a strong influence on subsequent incidental forgetting. Specifically, our results demonstrate that emotional forgiveness leads to substantially higher levels of forgetting in respect to offense relevant traits compared to both decisional forgiveness and no forgiveness.

While the biblical verse as well as the saying to “forgive and forget” suggest that the offense itself should be forgotten, our results yield that not the memory itself is impaired, given that the main effect of forgiveness on overall performance is not significant, but only forgetting of offense relevant traits is enhanced in the emotional forgiveness condition. By demonstrating that emotional forgiveness leads to forgetting of offense relevant traits the present study provides important evidence for cognitive aspects involved in the process of forgiveness. To our knowledge this is the first experimental evidence of this kind of relation.

When scrutinizing the results, one finds the difference between relevant and irrelevant traits in the decisional forgiveness and the control condition being close to significant, with those in the control condition showing a trend. Thus, participants in the decisional forgiveness and the control condition remember offense relevant traits somewhat better than offense irrelevant traits. This reverse pattern could be due to the fact that if an individual has not emotionally forgiven an offense, individuals still ruminate about the transgression and offense relevant traits are still even more salient as compared to non-offense relevant traits, which further supports our hypothesis that emotional forgiveness is a precondition to forget negative associations in respect to the transgressor.

Moreover, the present findings corroborate the proposition by Worthington and colleagues [22] that a behavioral intention to respond differently toward a transgressor (decisional forgiveness) differs substantially from the replacement of negative unforgiving emotions with positive other-oriented ones (emotional forgiveness). We are not suggesting that decisional forgiveness is not important step in the process of forgiving, however, regarding the process of forgetting our results show that there is no difference between decisional forgiveness and unforgiveness and hence indicate that decisional forgiveness has similar cognitive consequences as no forgiveness at all.

In the field of decision making, evidence is accumulating that emotions play a pivotal role in the decision making process [23,38] and that decision making may not even be possible or far from optimal without emotional involvement [24]. Similarly, the present study results yield that emotional forgiveness differs substantially from the mere decision to forgive. The fact that only those in the emotional forgiveness condition forget offense relevant traits is an indicator that complete forgiveness depends on emotional involvement.

In line with our results, Alexandra Asseily, a witness of the civil war in Lebanon (1975 to 1991), states in the documentary “The Power of Forgiveness” [39] that “Forgiveness allows us to let go of the pain and the memory. If we let go of the pain and the memory, we can have the memory, but it doesn’t control us.” This statement fits nicely to our proposition that it is not the offense itself that is forgotten but rather the responsibility attributions and as such the traits that are ascribed to the transgressor. Thus, we are not suggesting that one should just try to forget an incident, but rather free oneself from it. It has been shown that individuals, who ruminate about their depressive symptoms or anger, become more depressed or angry and stay that way for a longer period of time [40,41]. Similarly, when individuals ruminate about a transgressor they become more aggressive than when being distracted [42]. Thus, rumination may exacerbate responsibility attributions, which impedes forgiveness. This may cause a vicious circle of ruminating about a transgression, being unable to forget it, and consequently making responsibility attributions more salient to the victim.

At this point we can only speculate about the underlying affective and cognitive mechanisms driving the effect. One plausible explanation could be that the emotional significance is reduced through emotional forgiving making the offense relevant traits less salient and therefore less accessible in memory. Negative events and items can be remembered better and individuals better recall emotional items [43–46]. Thus, if a transgression that has not been entirely forgiven the negative traits, which are associated with the offense, may be more salient and thus will be remembered better.

Another plausible mechanism for the relationship between forgiving and forgetting may be a change in responsibility attributions. In line with this, a current study shows that individuals perceive an offense to be less controllable by the transgressor when they emotionally forgive the transgressor [47]. From an evolutionary perspective forgiveness serves the purpose of helping ancestral humans to get along with their genetic relatives and to establish and maintain cooperative relationships with nonrelatives [48]. Thus, forgiveness is suggested to be an important process for maintaining beneficial relationships. However, it also seems adaptive to remember painful experiences [8] to avoid similar hurts in the future. Taken together, it seems adaptive to forget not the hurtful experience itself but to change the attitude towards the offender. Typically, individuals, who are wronged, are inclined to make responsibility attributions about the offense. In line with that, Exline, Yali, and Lobel [49] found that when individuals were placed in the victim role, they were more likely to portray offenses as harmful, intentional, malicious, and less likely to judge them as justifiable. However, when attempting to maintain a beneficial relationship it seems counterproductive to blame the transgressor that hurts have been made intentionally and to remember these responsibility attributions and as such traits that are ascribed to the transgressor, who committed the offense. In line with that, Takaku [50] found that perspective-taking leads to benevolent attributions, benevolent emotional reactions, and forgiveness toward the transgressor. Thus, not the painful experience itself should be forgotten but the extent to which the offender is held responsible for a transgression should be changed. This may in turn lead to the fact that victims have less access to negative offense relevant traits of the offender and to forget these traits, which makes it easier to re-establish closeness to the offender.

Several limitations of the current study are noteworthy. First, subsequent work is needed to replicate the results in an independent highly powered sample to confirm the robustness of the effect. Another issue relates to the generalizability of the results. Given that there are much more female psychology students in our department, the present study was run in an all females sample and it is an open question if the findings generalize across gender. A recent meta-analysis [51] yielded that there is a mean effect size of gender differences in forgiveness of d = .28 indicating that females are more forgiving than males. Looking at moderating factors, Miller and colleagues found that those differences were more prominent in trait forgiveness (d = .30) than in state forgiveness (d = .23). Thus, the gender difference in a study inducing forgiveness and thus focusing on individuals’ states should be rather small. Moreover, a recent study found that while men reported more vengefulness than women in the control condition, men expressed similar or even lower levels of vengefulness than women when they were asked to either recall a similar offense of their own or consider the offender's perspective. Thus, men’s willingness to forgive should likewise be affected when they are asked to feel empathy towards the offender and as a consequence should lead to more forgetting of offense relevant traits. Moreover, in respect to memory performance in cognitive ability tests a meta-analysis by Stumpf [52] suggests that females show slightly better memory performance than males. Nevertheless a study by Cahill and colleagues [53] looking at amygdala participation in the storage of emotional material, did not find gender differences in the memory of neutral or negative emotional material. So, even if memory performance overall might be different for men and women, we would not expect differences in respect to the effect of forgiveness on forgetting of offense relevant traits. Finally, the present study merely included negative traits as stimulus material. However, it would be an intriguing question how forgiveness may affect memory performance of positive stimuli and to see if forgetting of positive stimuli may even be reduced in the emotional forgiveness condition.

The practical implications of our findings are especially relevant for interventions and psychotherapy designed to promote forgiveness. From a positive psychotherapy perspective individuals should seek to attend to and develop strengths rather than just minimize problems [54,55]. The present results emphasize that it is not enough to decide to forgive, but just like in the decision making process it is important to rely on one’s feelings to find optimal solutions (e.g., [24]), it seems important to incorporate emotions and empathy when truly forgiving someone. Thus, empathy for the transgressor and positive feelings toward the transgressor seem a core aspect when forgiving someone and an important component of forgiveness interventions.

In closing, little is known about the relationship between forgiving and forgetting. Our results provide first proof that emotional forgiving causes forgetting and will hopefully trigger research on cognitive aspects involved in the forgiveness process as well as research on the differentiation between various facets of forgiveness (e.g., emotional vs. decisional forgiveness.
